# Practices and Perceptions of Community Pharmacists in the Management of Atopic Dermatitis: A Systematic Review and Thematic Synthesis

**DOI:** 10.3390/healthcare11152159

**Published:** 2023-07-29

**Authors:** Abdi Berk Cayci, Adam Pattison Rathbone, Laura Lindsey

**Affiliations:** 1 School of Pharmacy, Faculty of Medical Sciences, Newcastle University, Newcastle upon Tyne NE1 7RU, UK; adam.rathbone@ncl.ac.uk (A.P.R.); laura.lindsey@ncl.ac.uk (L.L.); 2 Faculty of Pharmacy, Hacettepe University, Ankara 06100, Türkiye

**Keywords:** atopic dermatitis, community pharmacist, pharmacy practice, pharmacy education, systematic review

## Abstract

Understanding the contributions of community pharmacists as first-line health providers is important to the management of atopic dermatitis, though little is known about their contribution. A systematic review was carried out to examine practices and perceptions of the role of community pharmacists. A literature search was conducted in five different databases. Full-text primary research studies, which involved practices and perceptions of the role of community pharmacists in the management of atopic dermatitis, previously published in peer reviewed journals were used. Critical appraisal of included studies was performed using the Mixed Methods Appraisal Tool. Data were extracted and thematically synthesized to generate descriptive and analytical themes. The confidence of the findings of the included studies was assessed via either GRADE or CERQual. Twenty-three studies were included. Findings showed that community pharmacists lacked knowledge of the uses of topical corticosteroids. The recommendations of other treatments were limited. Pharmacists generally undertook dermatology training after graduation. Analytical themes indicated that the practices of community pharmacists were poor and misled patients. Inappropriate education in initial training was identified as a potential reason for their poor practices. This systematic review reveals a gap between patients’ needs in practice and dermatological education provided to community pharmacists. Novel approaches regarding education and training should be explored to improve pharmacists’ dermatological knowledge and skills.

## 1. Introduction

Atopic dermatitis (AD), which is also known as atopic eczema, is a skin disorder with a prevalence rate of 15–20% in developed countries [1]. It is usually accompanied by allergic rhinitis, asthma, and infection [2]. Although typically regarded as a childhood disease that presents before children reach one year of age, with the highest prevalence of onset being in 0–6-month-old infants [3], some patients (10–30%) may still have symptoms during adulthood [4]. AD is generally characterized by inflammatory flare-ups accompanied by acute (reddish and swollen) or chronic (lichenified) pruritic lesions on the skin [5]. According to streamlined and validated diagnostic criteria devised by the American Academy of Dermatology (AAD), essential features, such as pruritus, must be present for diagnosis. Some important features, such as an early age of onset, also support the diagnosis [6]. The clinical severity of AD is assessed based on the affected area and intensity using a standardized SCORAD tool [7]. As a recurrent chronic condition, AD affects patients’ physical health, financial circumstances, and quality of life [8].

Moisturizers are the mainstay of ongoing management of AD, and topical corticosteroids (TCs) are the first-line treatment for inflammatory symptoms and flare-ups. Topical calcineurin inhibitors (TCIs) are used in tandem with or as alternatives to TCs. In severe cases, systemic therapies, such as systemic corticosteroids, methotrexate, oral cyclosporine, dupilumab, and Janus kinase (JAK) inhibitors, are applied if first-line treatment fails [9,10]. Apart from these therapies, complementary and alternative medicines (CAMs), which are healthcare practices and products, such as herbal remedies, not commonly considered for use in conventional clinical medicine, have also gained popularity among patients with long-term AD who require alternative options to treat their condition [11]. However, these treatment options are used to control AD, decrease symptoms, and reduce flare-ups [12,13], and they cannot cure it. Therefore, creating a management plan is crucial to successful treatment [13].

AD symptom control requires treatment adherence. Poor understanding of the disease, forgetfulness, and the practicalities of applying topical medications have been identified as reasons for non-adherence [14]. Additionally, medications may be misused due to a lack of knowledge about fingertip units (FTU), which describe the quantity of TC to use per application, i.e., an amount from the fingertip to the first crease, that need to be applied to cover a body area the same size as a hand [15]. Also, corticosteroid phobia (corticophobia) is a common cause of treatment non-adherence [16], and it is based on patients’ belief that TCs are similar to anabolic or oral steroids [17]. It has also been shown that TCs may lead to adverse reactions, such as skin atrophy, striae distensae, rubeosis, and even adrenal insufficiency, after patients stop their long-term use [18,19]. Moreover, corticophobia could potentially develop in patients due to misinformation presented in the mainstream media [17]. Therefore, better AD symptom control could be achieved by helping patients to understand AD and how to use treatments by giving the right information to patients at the right time.

Since community pharmacists are first-line health providers [20,21], they play a key role in the management of AD, as patients often try over-the-counter treatment before seeking more urgent medical attention [22]. Pharmacists in some jurisdictions, like the UK and USA, can supply low-potency TCs, such as hydrocortisone of up to 1%, without prescriptions, although the majority of treatments are prescribed by specialists or doctors [23,24]. Little information exists regarding the ways in which pharmacists interact with patients regarding AD symptoms or management. More focused and coherent approaches can help pharmacists to support patients with AD, which may improve treatment outcomes. Appropriate counseling could help patients to overcome misinformation and increase adherence, improving symptom control. However, there is no systematic review of the literature regarding the contribution of community pharmacists to the management of AD. Therefore, this systematic review aimed to examine current practices and perceptions of community pharmacists in the management of AD discussed in the literature.

## 2. Method

### 2.1. Design

A mixed systematic review was carried out based on a convergent integration approach [25] that used evidence related to practices of community pharmacists and qualitative and quantitative methods.

### 2.2. Search Strategy

The review followed PRISMA [26] guidelines and was registered with PROSPERO (CRD42022308405). The search strategy, which is explained in the Supplementary Material document (Table S1), identified studies that investigated practices and perceptions of community pharmacists regarding the management of AD. Five databases (Ovid MEDLINE, Ovid EMBASE, EBSCO Cumulative Index to Nursing and Allied Health Literature (CINAHL), PsycINFO, and PubMed) were searched for data collected between their date of inception and December 2022. The search strategy was developed by the primary author (ABC), reviewed by other authors (LL, APR), and quality checked by a specialist librarian.

### 2.3. Selection Process and Inclusion Criteria

After the search, all identified articles were sent to Endnote. All titles and abstracts were reviewed by ABC to determine their eligibility. All authors assessed these titles against the inclusion criteria, which were determined in line with PICOS (population, intervention, control, outcome, and study design), as shown in Table 1. Inclusion criteria for studies were as follows: (i) research available in full-text form, (ii) primary research discussed in any language, (iii) research published in peer-reviewed journals, and (iv) research that investigated practices and perceptions of community pharmacists regarding the management of AD. Studies that did not fulfil the inclusion criteria were excluded.

### 2.4. Assessment of Methodological Quality

The methodological quality of the studies was appraised by ABC using the Mixed Methods Appraisal Tool (MMAT) [27] and reviewed by the remaining authors (LL, APR). Disagreements were solved via discussion. MMAT was used as it enables quantitative, qualitative, and mixed method studies to be critically appraised [28]. After two screening questions were asked, five questions remained, which participants could answer with either “yes”, “no”, or “can’t tell” responses. The results gave an overall assessment of methodological quality using either 0–1 (low quality), 2–3 (medium quality), or 4–5 (high quality) scores.

### 2.5. Data Extraction and Synthesis

Information (author and year, country, study design, methods of data collection, participants, number of participants, aim, key findings, and further recommendations of study) was extracted from the involved studies by ABC and reviewed by APR and LL. Data from qualitative and quantitative studies were synthesized using thematic synthesis [29], and a data-based convergent integration approach was applied [25]. Firstly, the quantitative data were subjected to a process known as “qualitizing” or “data transformation”, in which quantitative results were turned into textualized qualitative data [30,31]. This process was carried out by ABC, who converted summaries of statistical responses and author commentaries into descriptive textual data, which were then checked by APR and LL to synthetise quantitative data alongside the qualitative findings. Afterwards, the qualitized data and qualitative findings were synthesized via the following three-stage approach: (1) inductive line-by-line coding of findings acquired from themes, quotes, and author commentaries in qualitative studies and descriptive textual data in quantitative and mixed methods studies, as well as author commentaries; (2) combination of related codes into “descriptive” themes; and (3) generation of “analytical” themes based on the interpretation of the findings that went beyond the primary findings [29]. Initial coding and identification of descriptive themes were performed by ABC and reviewed by APR and LL. The agreed descriptive themes were combined into analytical themes through discussion until consensus was achieved among all authors.

### 2.6. Assessment of Confidence

The confidence of the synthesized findings obtained using qualitized descriptive quantitative and qualitative data was evaluated via the Confidence in the Evidence from Reviews of Qualitative Research (CERQual) tool [32], and findings derived from pre- and post-education intervention studies were assessed using the Grading of Recommendations Assessment, Development, and Evaluation (GRADE) approach [33]. The confidence was assessed using the CERQual tool based on four components—methodological limitations of the included studies, the relevance of included studies to the review question, the coherence of the findings, and the adequacy of the data used to support the review finding—and the GRADE tool was used to evaluate the following criteria: risk of bias, imprecision, inconsistency, indirectness, and magnitude of effect. Both tools judged the quality of evidence using “high”, “moderate”, “low”, or “very low” rankings.

### 2.7. Outcomes Assessed

The main outcomes assessed to examine practices and perceptions of community pharmacists were their knowledge, recommendations, attitudes, and experiences regarding the management of AD. Secondary outcomes included the perspectives of other individuals (healthcare providers, patients, and parents) on pharmacists’ practices.

## 3. Results

**Figure 1:**
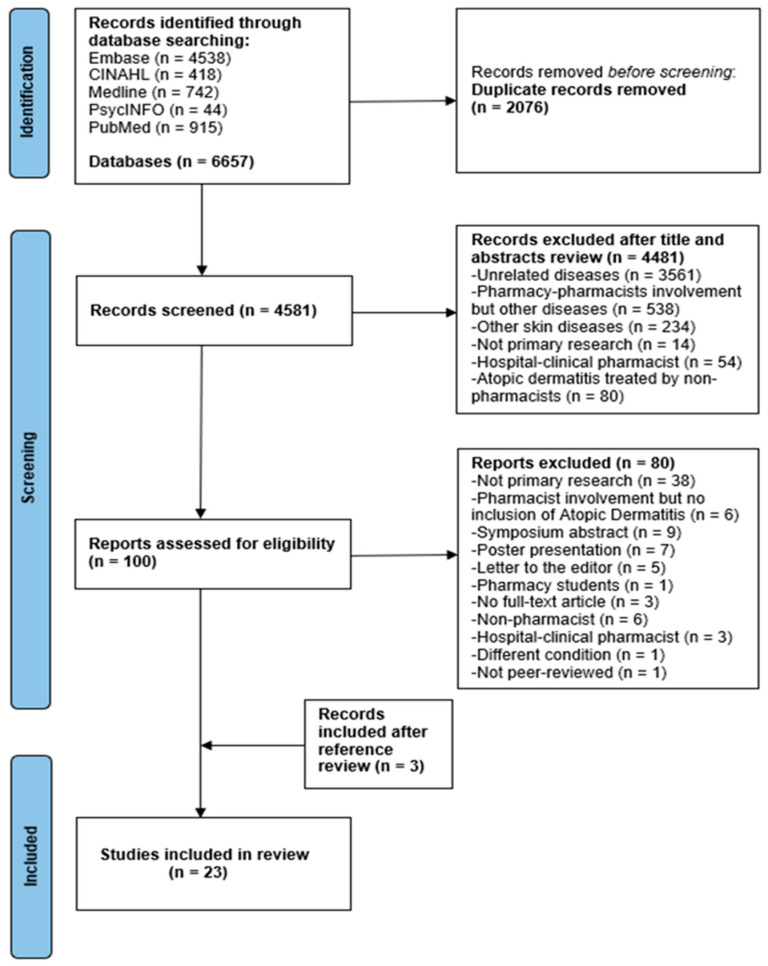
PRISMA flow diagram of search results and included studies.

A total of 6657 articles were identified. After removing duplicates and completing the screening stage, 100 studies remained and were evaluated for eligibility, and 80 studies were excluded. A further three studies were identified by searching the references of included studies. Finally, 23 studies (Figure 1), which were all published in English, except for one study published in Japanese, which was translated by a translator, between 1995 and 2021, were included for analysis.

Most studies (n = 19) were conducted in OECD countries: six studies were conducted in the United Kingdom [34,35,36,37,38,39]; two studies were conducted in each of Australia [40,41], Netherlands [42,43], Sweden [44,45], and Japan [46,47]; and one study was conducted in each of Portugal [48], France [49], Germany [50], Belgium [51], and Italy [52]. Moreover, one study was conducted in a GCC (Gulf Cooperation Council) nation, i.e., the United Arab Emirates [53]. Studies were also carried out in three less developed countries: Jordan [54], South Africa [55], and Iraq [56]. No studies were conducted in the least developed countries.

A majority of studies (n = 20) were published in the period 2011–2021 and reported quantitative findings (n = 18). Of these studies, 14 were cross-sectional studies [37,39,40,46,47,48,49,50,51,52,53,54,55,56] and 4 were pre- and post-education intervention studies [34,41,43,44]. Three were qualitative research [35,42,45]. The remaining studies [36,38] used mixed methods. Table A1 shows a summary of the included studies (see Appendix A section).

The results of quality assessment are shown in Table S2. There was much diversity in scores across studies, which ranged between low and high quality. Only one study was denoted as being of low quality [56]. The quality scores of the quantitative studies ranged from low to high quality, with 13 of 18 studies ranked as being of medium quality. All qualitative studies were ranked as being of high quality [35,42,45]. Two mixed methods studies were rated as being of medium quality [36,38]. Common issues related to study quality and risk of bias for quantitative and mixed methods studies were unclear descriptions of the target population and the sample, as well as well-described inclusion and exclusion criteria for the sample. The main issue that affected qualitative and mixed methods studies was a lack of clarity regarding the way in which the findings were derived from the data.

### 3.1. Descriptive Themes

**Figure 2:**
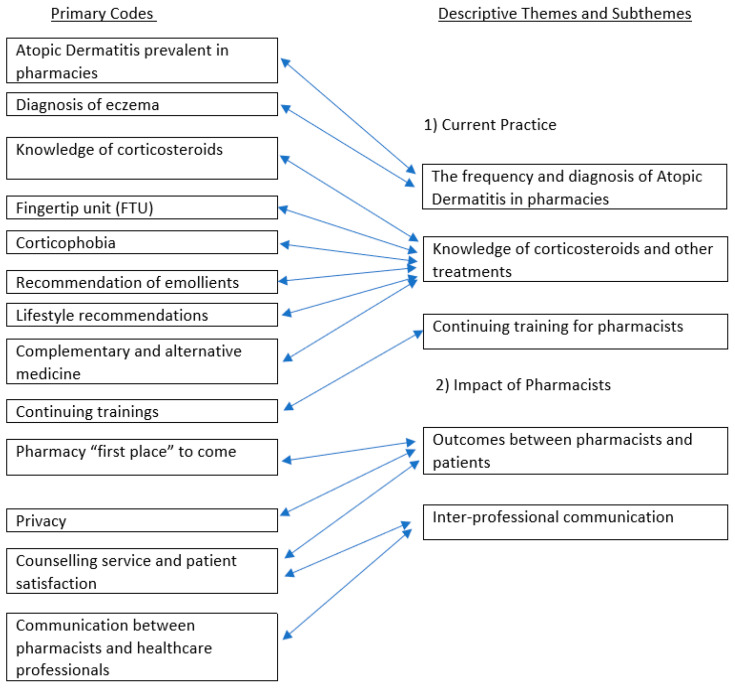
Codes, sub-themes, and descriptive themes.

Synthesized statements derived from descriptive quantitative and qualitative data were produced, and the confidence level of each statement was assessed using the CERQual tool (see Table S3). Other statements from pre- and post-intervention studies were evaluated using GRADE. As the designs of experimental intervention studies were evaluated as poor, the general rating of findings was reduced from low to very low quality [57]. Two descriptive themes and their sub-themes were identified via the analysis by combining relevant codes (Figure 2; Theme 1: Current Practice and Theme 2: Impact of Pharmacists). The descriptive themes and sub-themes are outlined below.

#### 3.1.1. Current Practice

- Knowledge of Corticosteroids and Other Treatments

Knowledge of corticosteroids was a significant theme, having FTU- and pharmacist corticophobia-related sub-themes, and it was mentioned in 14 studies [36,41,42,43,44,46,47,48,49,50,51,52,53,54]. Pharmacists were shown to lack knowledge of TC potency in the literature (CERQual-high) [36,53,54]. Poor knowledge regarding the length of TC use was also reported (CERQual-moderate) [48,49,51,54].

Fingertip unit (FTU):

Most pharmacists did not use standard measures, such as FTU, to communicate dosing instructions to patients in the literature. Instead, the literature indicated that patients were told to apply doses thinly (CERQual-moderate) [42,48,54]. Two more studies also stated that a tiny minority of pharmacists recommended FTU to patients (CERQual-moderate) [47,51].

Similarly, pharmacists tended to tell patients to “apply it sparingly” in Australia, though they began using FTU after an educational intervention (GRADE-very low) [41]. It was also found that although most pharmacists knew the amount that can be measured via FTU, only the minority of them often or always advised patients to use this method (CERQual-moderate) [36,47].

Pharmacist corticophobia

Corticophobia was identified in the literature among pharmacists (CERQual-high) [36,42,43,49,51,52]. A Belgian study that compared practitioners found that corticophobia was higher among pharmacists than paediatricians, general practitioners, and dermatologists (CERQual-low) [51]. In France, pharmacists’ average confidence regarding corticosteroid use was rated as medium (CERQual-low) [49]. Pharmacists were confused about the side effects of oral corticosteroid and TC use, and they thought that TCs may cause systemic effects (CERQual-moderate) [49,51]. However, it was shown corticophobia can be alleviated via educational intervention (GRADE-very low) [43].

Other treatments, including emollients, lifestyle habit changes, TCIs, and CAMs, were also mentioned [36,42,44,46,54,55]. Regarding emollients, most pharmacists recommended using them as an initial treatment (CERQual-moderate) [36,54], even if they were not prescribed by doctors (CERQual-low) [42], and patients were advised by pharmacists to use them regularly for a prolonged period (CERQual-low) [36]. CAM use was mentioned in only one study [55] and CAMs were seen as more comprehensive and beneficial treatments than current treatment by some pharmacists (CERQual-low). Recommendation of TCI use was mentioned in one study, in which pharmacists explained that a tingling sensation is a common side effect about which patients should not worry (CERQual-low) [46]. Another study noted that pharmacists rarely gave recommendations about lifestyle habits to patients (CERQual-low) [42].

ii. The Frequency and Diagnosis of Atopic Dermatitis in Pharmacies

AD is one of the most prevalent skin conditions seen in pharmacies (CERQual-moderate) [37,39,40,48]. Community pharmacists carry out more medicine reviews for eczema than any other skin condition in the UK (CERQual-low) [39].

Two studies evaluated the diagnostic ability of pharmacists using expert assessors [38,40]. Although some assessors concurred with the diagnoses determined by pharmacists, medical history-recording behaviors of pharmacists were found to be inadequate (CERQual-low) [38]. Furthermore, only in 67% of cases diagnosed as AD did a dermatologist agree with a pharmacist’s decision (CERQual-low) [40].

iii. Continuing Training for Pharmacists

Many studies demonstrated that pharmacists often continued their dermatology education after graduation (CERQual-moderate) [36,37,39,40,49,52,53,55]. Pharmacists were eager to expand their dermatological expertise, which they acquired by joining educational programs or training sessions held by drug manufacturers, attending conferences and branch meetings, or reading journal articles and e-bulletins (CERQual-moderate) [35,36,37,49]. There was a strong correlation between the extent to which continuing their training in dermatology helped pharmacists and their overall self-confidence (CERQual-low) [39]. Pharmacists who undertook continued dermatology training displayed better knowledge, attitude, and practices regarding TC treatment (CERQual-moderate) [53].

#### 3.1.2. Impact of Pharmacists

- Outcomes between Pharmacists and Patients

Three sub-themes were identified in the literature as being associated with outcomes between pharmacists and patients: “pharmacy first place to come”, “counseling service”, and “privacy” [34,37,38,41,43,44,45,52,54,56]. Some studies placed emphasis on pharmacies as the first place that patients visit upon developing a skin problem (CERQual-high) [37,45,52,54], though patients were referred to doctors if they had flare-ups or the condition deteriorated (CERQual-moderate) [45,54].

Counseling services by pharmacists were also reported [34,37,38,43,44,52,56], and most patients were satisfied with the service (GRADE-very low) [34,43], as were pharmacists (GRADE-very low) [44]. However, in a study conducted in Iraq, most patients did not receive any information about the use and adverse effects of TCs from pharmacists (CERQual-very low) [56].

Patient privacy was not usually a concern for pharmacists [41,45]. Pharmacists held consultations with patients in front of other people (GRADE-very low) [41], and patients reported feeling agitated during the consultation [45]. Furthermore, patients reported that pharmacists were not able to understand patients’ circumstances (CERQual-low) [45].

ii. Inter-professional Communication

In the literature, communication and collaboration between pharmacists and healthcare professionals was weak (CERQual-moderate) [35,45,49,50]. For example, different guidelines were used by healthcare professionals, with no synchronized approach used, and this issue may cause confusion for patients (CERQual-low) [35]. Moreover, the duration of use of TCs prescribed by physicians was mostly decreased by pharmacists after plausibility checks (CERQual-moderate) [49,50].

### 3.2. Analytical Themes

Through the analysis of descriptive themes, two analytical themes were determined, which sought to go beyond the findings reported in the original study [58]. The analytical themes discovered were “misleading position” and “perceptions of education and training”.

#### 3.2.1. Misleading Position

Pharmacists potentially misinformed patients regarding knowledge of and recommendations and practices regarding AD and its treatment. Although AD was a skin condition commonly seen in pharmacies, while community pharmacists considered themselves to be first-line providers of treatment to patients with dermatologic conditions (Quote 1), they may misinform patients using TCs because of their insufficient knowledge about the potency of TCs (Quote 2).

“*Pharmacists should be the first port of call for patients with a skin problem*”.

[37] Quote 1

“*In terms of formulations, over 60% did not know how many topical corticosteroid potency categories exist*”.

[36] Quote 2

Besides insufficient knowledge of TCs, pharmacists lacked a standardized way of communicating advice to patients who used topical treatments. Rather than using FTU, they recommended thinly applying topical medications (Quote 3).

“*Of course, you have those fingertip units. Well, I must confess that we don’t really work with it to indicate how much you have to apply. We just say: apply thin. It is still a hormone cream*”.

[42] Quote 3 (Pharmacist)

Moreover, different terms, besides “applying thinly”, were used by pharmacists, which may make patients more confused and worsen existing corticophobia. Subsequently, this situation may result in treatment non-adherence.

“*When directing the amount of TCS to be applied, 54% reported informing the patient that TCS should be used sparingly…”*

[41] Quote 4

Despite AD being one of the most commonly encountered conditions in pharmacies, the pharmacists’ ability to record medical histories in some cases was poor (Quote 5). This issue may lead to patients being misinformed about the proper use of treatment or cause misdiagnosis.

“*A more detailed history would have been helpful and may have supported making the diagnosis*”.

[38] Quote 5 (Dermatology Specialists)

Regarding emollients, although pharmacists advised patients to apply emollients, even if they were not prescribed (Quote 6), they did not take into consideration the utility of tailored moisturizers for different skin types, sensitivities, or allergies.

“*Maybe they weren’t told about the emollient at the GP. And then you give the advice to use a moisturizer*…”

[42] Quote 6 (Pharmacist)

Overall, with moderate-to-high confidence, the evidence suggests that pharmacists were misinforming patients about AD management in practice. With lower confidence, findings also suggested poor diagnosis by pharmacists. Therefore, we can conclude with moderate confidence that community pharmacists may inadvertently play a role in patients being misdiagnosed, inaccurately using treatment, or using an insufficient amount of medicine.

#### 3.2.2. Perceptions of Education and Training

The literature showed that pharmacists received postgraduate training in dermatology, and most appeared to be satisfied with these educational tools. Besides training, pharmacists and their teams used educational interventions for the treatment of AD, which helped them to improve their treatment practices (Quote 7,8).

“*Of those (pharmacists) surveyed, 92% stated they would advise TCS be used until the eczema is clear, compared to 27% prior to education (p < 0.0001)*”.

[41] Quote 7

“*Knowledge about eczema and treatment among pharmacy staff increased from baseline to follow-up 7.3 ± 1.7 to 8.4 ± 1.5 (p = 0.052)*”.

[43] Quote 8

Furthermore, the pharmacists who attended educational training sessions or interventions could better counsel patients, which resulted in improvements in the management of the condition (Quote 9).

“*Also, parents were, in general, positive about the counseling session in the pharmacy […] 45.8% mentioned they started using the treatment differently afterwards (e.g., more frequent use of emollients and increased application of TCS, based on FTU)*”.

[43] Quote 9

Considering the educational level of community pharmacists regarding dermatology, further educational training is perceived as effective at improving practice and patient care. However, in the literature, current practice of pharmacists was poor, which may be caused by the fact that knowledge acquired via initial education alone might not be enough to ensure good quality care in practice. Hence, the gap between pharmacists’ knowledge and the needs of the patients can be addressed by improving dermatological education in the initial training of pharmacists.

Overall, with moderate confidence, the evidence showed that pharmacists were willing to extend their knowledge through further education, though the confidence of findings regarding the effectiveness of educational interventions was very low.

## 4. Discussion

### 4.1. Summary of Findings

This study reviewed the literature regarding the practices and perceptions of community pharmacists in the management of AD. A key treatment recommended by pharmacists was TCs, though only a few studies mentioned their knowledge and practices regarding other treatments. The most striking finding to emerge from the analysis is that pharmacists did not effectively communicate information about TCs to patients. Pharmacists lacked knowledge of TC practice and duration of treatment. In addition, they had corticophobia. Although most pharmacists knew about FTU, they did not use it. Even though “apply thin” has been removed from labels and the FTU has been promoted in the Netherlands since 2013 [59], this practice was retained by pharmacists in 2019 [42].

Regarding corticophobia, some pharmacists exhibited more fear of using TCs than other healthcare professionals, and this situation encouraged patients to be suspicious of TCs. A key finding is that a pharmacist’s stance on TCs may mislead patients regarding their effective use and treatment. A similar position was offered by Smith et al., who reported that cautious approach preferred by pharmacists may encourage patients to avoid TCs [60]. Subsequently, this situation may lead to the ineffectiveness of therapy, since corticophobia is already quite high in patients with AD from 15 different countries [61] and seen as one of the main reasons for non-adherence [16]. It cannot be denied that corticophobia is common in patients; however, it is important for pharmacists to provide accurate and unbiased information and support to patients with AD.

Another key finding is that pharmacists could provide better AD support in practice if they received more comprehensive education on dermatology. It was shown that the counseling practice of community pharmacists was affected by their level of education [62]. This finding is supported by a previous study, which found that re-education of pharmacists was a potential way to enhance confidence in the treatment provided [60], especially as the ability to address dermatological questions has been found to be low among pharmacy students [63]. In addition, receiving educational training was linked to confidence in dealing with skin conditions [64]. Furthermore, an e-learning educational program that included corticophobia and FTU sections was implemented in a past study, and an increase in the knowledge of AD management was observed in pharmacists [65].

### 4.2. Implications for Practice and Policy

While community pharmacists play a key role in counseling patients with AD, they lacked practical knowledge of the management of the condition. Pharmacists could negatively influence patients by advising incorrect ways of using topical treatments, preventing proper use of TCs and potentially causing misdiagnosis of conditions. The findings also showed that pharmacists attended a range of training courses related to dermatology after graduation. There is an evident gap between patient needs in pharmacy practice and initial education. This gap can potentially be reduced via educational interventions, such as giving pharmacy students sufficient knowledge of the use of TCs and other topical treatments and providing more comprehensive competency-based practice education regarding dermatology. If patients are not appropriately helped to manage their condition by pharmacists, they may continue their usual routine, and the condition may remain unmanaged. An increase in the number of unmanaged AD patients may put more financial burden on countries’ health systems, since AD has a significant financial effect on health care systems [66]. To avoid this burden, preventative action can be taken by policymakers and academics. Providing dermatological training during initial pharmacy education should help pharmacists to develop the improved knowledge and skills required to meet patients’ needs in practice. This training should draw on expertise from dermatologists, patient experts, and practices that are captured in current guidance [23,67,68]. This training needs to be ongoing and embedded in practice through continuous professional development to ensure that pharmacists can appropriately assess, diagnose, prescribe, and monitor AD, as well as communicate with patients, in community pharmacy settings.

### 4.3. Strengths and Limitations of the Study

The strength of the study is that it identified, for the first time, the current strengths and weaknesses of the practices used by pharmacists to supporting patients with AD. Regarding limitations, TC was the most commonly mentioned medication in this review, though it does not give a full picture of counseling practices used by pharmacists for all treatment options. Some studies notably concentrated on paediatric eczema, though eczema is not seen in children alone. Some included studies discussed other skin conditions besides AD, and this issue may limit these studies’ findings’ of relevance to AD. In addition, all studies were derived from either OECD, GCC, or less developed countries; thus, the findings of this study may not be applicable to least developed countries. Some studies cited other healthcare professionals as well as pharmacists, and it was possible to separate the findings attributed to pharmacists in these studies. However, in two studies [43,44], pharmacists and technicians, and in one study, [46] community and hospital pharmacists were grouped together as pharmacy staff, meaning that distinguishing the pharmacists’ contributions was not possible in these studies.

### 4.4. Further Research

Further studies are needed to broaden this topic’s focus to cover other treatments of AD, rather than only studying TCs. The focus of future pedagogical research needs to be on establishing steps that can be taken as part of initial education to improve practice of future pharmacists in supporting patients with AD. Based on the low confidence rates presented in some studies, more robust research is needed. There is also a gap in evidence regarding pharmacist management of AD in least developed countries.

## 5. Conclusions

The purpose of the study was to examine community pharmacists’ contribution to the management of AD. This study has shown a gap between community pharmacy practice and pharmacists’ training and education. This gap means that pharmacists inadvertently mislead patients in practice by reinforcing fear of TC use and providing inadequate counseling about the duration and application of topical treatment. Despite significant receiving undergraduate and postgraduate training, the literature indicates that community pharmacists lacked the knowledge and skills required to effectively respond to the needs of AD patients in practice, encouraging them to seek further dermatology training to make up for the educational deficiency of their initial training. The findings of this research provide insights into the gap between practice and education, showing that novel educational interventions are required to improve AD management.

## Figures and Tables

**Table 1:** PICOS.

**Population**	Patients with atopic dermatitis (all ages)
**Intervention**	Practices and perceptions of community pharmacists in the management of atopic dermatitis
**Control**	None
**Outcome**	Main: Pharmacists’ knowledge, recommendations, attitudes, and experiences regarding the management of atopic dermatitis Secondary: Perspectives of others (healthcare providers, patients, and parents) regarding the practices of pharmacists
**Study design**	Qualitative, quantitative, and mixed methods studies

## Data Availability

Data that support the reported findings of this study are found in the Ovid MEDLINE, Ovid EMBASE, EBSCO Cumulative Index to Nursing and Allied Health Literature (CINAHL), PsycINFO, and PubMed databases.

## Supplementary Materials

The following supporting information can be downloaded at: https://www.mdpi.com/article/10.3390/healthcare11152159/s1, Table S1: Detailed search strategy; Table S2: Quality assessment of included studies; Table S3: CERQual full evidence profile; File S1: PRISMA 2020 checklist. Refs [26,34,35,36,37,38,39,40,41,42,43,44,45,46,47,48,49,50,51,52,53,54,55,56] are cited in Supplementary Materials.

## Appendix A

**Table A1:** Summary of included studies.

Author and Year	Country	Study Design	Methods of Data Collection	Participants	Number of Participants	Aim	Key Findings	Further Recommendations
Abed et al. [56], 2021	Iraq	Cross-sectional	Questionnaire	Customers who asked for topical corticosteroids (TCs) without prescription	212 customers	To assess the patients’ knowledge of TC use and education provided by pharmacists	The lack of pharmacist practice regarding patient education	Health officials should raise awareness of incorrect use of TCs by advertising in public and via traditional and social media.
Carr et al. [34], 2009	England	Pre-post educational intervention	Questionnaire	Patients and their parents	50 children and their parents	To identify the effects of community pharmacists’ interventions on the use of emollients in children with eczema	Pharmacists’ interventions reduced some symptoms to a moderate yet significant extent. In addition, the intervention taught patients to apply emollients properly. Although intervention was found to be useful, it requires pharmacists that devote a lot of time; thus, s new pharmacy regulations may make it easier for pharmacists to counsel patients.	The small number of pharmacies referenced in this study may not reflect the aspect of all pharmacists and some of them in this study have experience of offering counseling services. Therefore, this study may give inaccurate outcomes. To conclude, a greater number of pharmacies should be included for next studies.
Cowdell et al. [35], 2019	England	Qualitative study	Field notes and interviews	Community pharmacists and other health professionals	2 community pharmacists	To create positive atopic dermatitis (AD)-related mindsets among healthcare practitioners to improve management of the condition	Eczema was seen as simple condition to treat by practitioners, and they thought eczema treatment has changed little over time. Therefore, they did not need to improve their knowledge. However, pharmacy staff increased their knowledge through formal and informal sources.	Outdated mindsets should be removed by introducing reliable and beneficial knowledge. Improving mindsets is crucial for self-management of AD.
Giua et al. [52], 2021	Italy	Cross-sectional	Questionnaire	Community pharmacists	154 community pharmacists	(1) To obtain information about pharmacists’ counseling activity regarding dermatological conditions; (2) to gain information about corticophobia among pharmacists; (3) to research the educational needs of pharmacists	AD was the most known of all dermatitis types by pharmacists (79.9%). Pharmacists generally provided counseling services to all patients at least once a week. A majority of pharmacists (57.1%) believed that patients visit pharmacies before seeing a doctor. Although most of pharmacists (66.9%) thought that patients were adherent, they underestimated corticosteroid phobia (corticophobia) between patients, as only a minority of them had positive opinions about topical corticosteroids.	Pharmacists think that educational tools would be beneficial to solve the knowledge gap.
Hammarstrom et al. [44], 1995	Sweden	Pre-post educational intervention	Questionnaire and drug sale statistics	Community pharmacies	900 community pharmacies	To improve treatment management in patients with skin disorders	Almost all pharmacy staff agreed that input from local pharmacies in improving treatment of skin disorders was substantial. Both over-the-counter (OTC) and prescribed medication use significantly increased after campaign was held. The highest increase was recorded regarding the use of emollients. The increase in use of corticosteroids resulted from the campaign in which pharmacists emphasized importance of corticosteroid use in the management of AD. It was obvious that a transition from prescribed to non-prescribed medication use was seen after the campaign.	It is important to introduce more campaigns among community pharmacies.
Issa et al. [54], 2016	Jordan	Cross-sectional	Questionnaire	Community pharmacists	100 community pharmacists	To evaluate the disagreements in TCs prescription patterns and practice advices among different health workers and determine underlying causes	Pharmacists lacked knowledge about the potency of topical corticosteroids. Most pharmacists (67%) recommended “apply thin” instead of using “fingertip unit (FTU)” regarding use of topical corticosteroids. Despite the fact that a large majority of pharmacists (68%) recommended that patients see doctor in case of flare-ups, 28% of them still suggested the use of mid-high- or high-dose corticosteroids. Just over a third of pharmacists (36%) suggested the use of emollients for patients with mild eczema.	It is important to update pharmacists regarding practice guidelines to develop effective AD treatment.
Jairoun et al. [53], 2020	United Arab Emirates	Cross-sectional	Questionnaire	Community pharmacists	772 community pharmacists	To find out pharmacist knowledge, attitude, and practice in use of corticosteroids	Pharmacists with more experience had better knowledge and used better practices regarding corticosteroids. Pharmacists who received educational training had better questionnaire scores than those who did not receive training. There were knowledge, attitude, and practice differences between those who graduated from international and local institutions. There was low score in terms of use of potent corticosteroid in cases of acute flare ups of eczema. Many pharmacists thought that corticosteroids should not be used in children.	Proper courses regarding the appropriate use of corticosteroids should be integrated into the curriculum to educate pharmacists.
Kaneko et al. [46], 2014	Japan	Cross-sectional	Questionnaire	Community and hospital pharmacists	372 community, 109 hospital pharmacists	To investigate pharmacist practices regarding applying topical medications in the management of AD	Pharmacists mainly explained the best application site to patients regarding use of corticosteroids if they were using it first time, and pharmacists’ knowledge of guidelines positively affected their recommendations. Pharmacists urged patients to apply topical medications until their situation improved. Pharmacists gave information about tingling effects associated with the use of calcineurin inhibitors and told patients that this issue is common side effect. However, this approach caused anxiety in some patients. Pharmacists complained that doctors’ instructions regarding the frequency and site of application were not clear.	To effectively counsel eczema patients, pharmacists should be aware of treatment guidelines for AD and are urged to follow these guidelines.
Koster et al. [42], 2019	Netherlands	Qualitative study	Interviews	Parents of children with AD, community pharmacists, and pharmacy technicians	29 parents, 6 community pharmacists, and 12 pharmacy technicians	To explore the perspectives of both pharmacy staff and parents regarding the treatment of children with AD in the Netherlands	Pharmacists thought patients suddenly gave up corticosteroid, which was caused by the fact that pharmacy staff give misinformed information about corticosteroid use, which led to corticophobia in patients. Pharmacists did not use FTU to indicate the amount of cream that patients should apply. They simply told patients to thinly apply the cream. Pharmacists recommended the use of emollients in addition to treatment, even if they were not prescribed. Parents of children with AD needed additional information about lifestyle habits from pharmacists.	The problem of insufficient practical skills and knowledge about AD among pharmacy staff needs to be rectified.
Koster et al. [43], 2021	Netherlands	Pre- and post-education interventions	Questionnaire	Parents of children with AD, community pharmacists, and pharmacy technicians	48 parents, 6 community pharmacists, and 13 pharmacy technicians	To study the effects of pharmacy intervention on corticophobia among both pharmacy staff and parents of young AD patients	Educational interventions may be useful to overcome corticophobia, which is measured via the TOPICOP scale, for both pharmacy staff and parents of children with AD. The corticophobia score of pharmacy staff significantly decreased from 33.2 to 25.1% between pre- and post-intervention test. Knowledge of pharmacy staff about eczema and treatment rose after educational intervention. Parents of patients were happy with counseling serviced and began using medicines more regularly. Patients’ eczema conditions improved substantially after they received counseling services.	The follow-up time was short, meaning that it could only provide information about short-term effects of the intervention. Pharmacy staff should be regularly trained to provide optimal treatment to patients.
Lambrechts et al. [51], 2019	Belgium	Cross-sectional	Questionnaire	Pharmacists, paediatricians, GPs, dermatologists	118 pharmacists, 100 paediatricians, 81 GPs, and 92 dermatologists.	To determine the frequency of corticophobia among pharmacists and other health professionals	Pharmacists made up the group with the highest rate of corticophobia all other health professionals. More than half of pharmacists (55.1%) had proper knowledge of the amount that should be applied, and about half of them (48.3%) knew that a corticosteroid medicine must be used until eczema disappears. A minority of pharmacists (21.2%) recommended FTU as the best method with regard to the use of TCs.	Corticophobia among health professionals remains an issue because of insufficient knowledge, meaning that staff should be further trained, as approximately one-third of pharmacists do not remember receiving courses about TCs
Lau et al. [36], 2017	England	Mixed-methods	Interviews and questionnaire	Community pharmacists	5 community pharmacists were interviewed and 105 community pharmacists filled out the questionnaire.	To investigate the knowledge of community pharmacists regarding corticosteroid use in the treatment of AD, as well as their information supply, attitudes, and patient counseling behavior	Some pharmacists (36.2%) received extra postgraduate training related to dermatology. Most pharmacists (62.9%) inaccurately categorized corticosteroids in terms of their potency. Regarding corticosteroids, a minority of pharmacists stated that if patients use them correctly, side effects are not common. Though most of them (90.5%) knew about FTU, only a third of pharamcists (36.2%) always or often recommended this method when counseling patients. Pharmacists had more knowledge of the use of emollients than corticosteroids.	Pharmacists perceptions of corticosteroid safety are low. They need to be improved to ensure that they can effectively counsel patients.
Lindblad et al. [45], 2006	Sweden	Qualitative	Focus groups	Patients and health providers (community pharmacists, dermatology nurses, and dermatologists)	12 patients and 12 health providers (the number of pharmacists was not applicable)	To determine the views of health providers and patients regarding the role of providers in the management of dermatological conditions	If patients have not yet seen a doctor and do not know about their situation, pharmacists gave them basic information about eczema and some weak corticosteroids. Health providers labelled pharmacists as having “initial screener” and “final checker” roles. However, there is a lack of collaboration between pharmacists, nurses, and doctors. Patients thought that pharmacists lacked understanding of their situations and claimed that there was lack of privacy in pharmacies, and they did not feel able to communicate with pharmacists.	There is a need for successful cooperation between pharmacists and other health providers. Pharmacists suggest that regular meetings can be held regarding finding medicine information to settle the conflict. In addition, some strategies should be implemented to embed dermatology in healthcare systems.
Manahan et al. [40], 2011	Australia	Cross-sectional	Questionnaire	Community pharmacists and pharmacy interns	17 community pharmacists and 3 pharmacy interns	To identify community pharmacists’ roles in the management of skin conditions and assess their opinions of teledermatology services	Almost half of cases managed by pharmacists were eczema. In 2/3 of cases diagnosed as eczema, a dermatologist completely agreed with the pharmacist’s management of the condition. All pharmacists were keen to received further education related to dermatology.	A system of pharmacist–tele dermatologist cooperation could improve the management of diseases and alleviate the burden on GPs.
Oishi et al. [47], 2019	Japan	Cross-sectional	Questionnaire	Community pharmacists	300 community pharmacists	To investigate the effectiveness of community pharmacists’ instructions regarding the use of FTU and the effects of following practice guidelines on treatment	A small minority of pharmacists (14.3%) always used FTU to the primary method of corticosteroid application. The level of comprehension of best practice guidelines correlated with pharmacists’ ability to give instructions about topical corticosteroid use. Though pharmacists considered corticosteroid application amounts and sites, the timing of doses was ignored.	Community pharmacists must be acquainted with best practice guidelines to ensure that they can counsel patients about their treatment.
Raffin et al. [49], 2016	France	Cross-sectional	Questionnaire	Community pharmacists, technicians and students	176 community pharmacists, 10 pharmacy technicians, and 5 pharmacy students	To evaluate corticophobia among pharmacists in relation to AD in children	Among pharmacists, there was lack of knowledge about side effects of corticosteroids admiistered via topical and oral applications. Pharamcists had a lack of information about duration of corticosteroid treatment. Opposing instructions from pharmacists and physicians can cause confusion and fear in patients. A vast majority of pharmacists wanted to receive education about AD in children.	Continuing training for pharmacists regarding the use of corticosteroids is recommended to improve adherence results.
Salzmann et al. [50], 2020	Germany	Cross-sectional	Questionnaire	Community pharmacists and dermatologists	351 community pharmacists and 53 dermatologists	To acquire data about daily prescription habits related to compounded preparations (CPs) in dermatology and compare them using standardized questionnaires, as well as to overcome the lack of interdisciplinary collaboration	Pharmacists viewed compounded preparations as an “important treatment alternative”. There was inadequate communication and collaboration between pharmacists and dermatologists.	Future studies should concentrate on CPs to increase the quality of prescriptions.
Smith et al. [41], 2016	Australia	Pre-post educational intervention	Survey	Pharmacists (including community pharmacists)	292 pharmacists	To evaluate pharmacists’ beliefs and knowledge regarding the use of topical corticosteroids in paediatric eczema	The proportion of pharmacists who talked to patients separately for privacy reasons significantly increased from 41 to 78%. The share of pharmacists who recommended applying treatments as shown on prescription rose from 52 to 88%. The proportion of those who suggested corticosteroid use until eczema cleared up rose to 92%, and those who suggested a maximum use period of one week declined to 2%. The percentage of pharmacists who told patients to apply treatments sparingly fell to 8%.	Pharmacist education throughout initial education and pharmacy journals should be supported by dermatology specialists to enhance pharmacists’ knowledge of the use of TCs in AD.
Teixeira et al. [48], 2021	Portugal	Cross-sectional	Questionnaire	Community pharmacists and patients	149 community pharmacists, 44 patients	To find the association between pharmacists’ knowledge and their conveyance of information to patients with dermatoses and create a proper guide for patients about dosage instructions	Atopic dermatitis (84.3%) was the most prevalent condition in Portugal according to pharmacists. 63.9% of pharmacists thought that they always provided information about the duration of treatment with topical corticosteroids. 50% of pharmacists always instructed patients on how to apply medicine, and 85.2% of phramacists instructed patients on how to “apply in thin layer” while applying a topical medicine.	Proper guidelines must be prepared to enhance communication of dosage instructions to patients. Moreover, continued training courses should be implemented to help pharmacists to solve problems.
Thandar et al. [55], 2019	South Africa	Cross-sectional	Questionnaire	Community pharmacists	82 community pharmacists	To identify community pharmacists’ attitudes and practices regarding the use of complementary and alternative therapies in patients with AD	More than a third of pharmacists (35%) thought that complementary and alternative medicines (CAMs) outweighed conventional therapy in terms of effectiveness. Almost all pharmacists supported including CAMs in university curriculums. Although most pharmacists were not acquainted with homeopathy and Chinese herbal medicine, they were mostly familiar with probiotics.	It was revealed that pharmacists had insufficienct knowledge of CAMs, meaning that they needed further ongoing education.
Tucker et al. [37], 2012	England and Wales	Cross-sectional	Questionnaire	Community Pharmacists	870 community pharmacists	To find out which types of skin diseases pharmacists come across in pharmacies and which training sessions they attend to gain more knowledge of dermatological diseases	Eczema was the most commonly encountered condition for which patient want advice, besides dry skin complaints. Most pharmacists (64.8%) received some postgraduate training related to dermatology. Pharmacists were aware that they have critical roles in counseling patients and should be the first point-of-contact for patients with skin problems.	Use of pharmacists’ advice in the management of skin conditions should be studied in future studies.
Tucker et al. [39], 2013	England and Wales	Cross-sectional	Questionnaire	Community Pharmacists	870 community pharmacists	To identify pharmacists’ roles in medicine use review (MUR) and evaluate pharmacists’ understanding of long-term skin diseases	The most commonly conducted MUR services were in performed patients with eczema. Pharmacists mostly found postgraduate training to be helpful in terms of dealing with patient requests, and this willingness was positively associated with overall self-confidence.	Further studies should be conducted to identify whether these reviews contribute to better disease-related outcomes.
Tucker et al. [38], 2017	England	Mixed methods	Questionnaire- and assessment-based feedback	Patients, dermatology specialists, and community pharmacists	40 patients, 3 dermatology specialists, and 9 community pharmacists	To assess the clinical convenience of pharmacists’ diagnoses and management of dermatitis and acne, as well as to obtain patients’ self-reported perceptions regarding the effectiveness of pharmacy intervention	Pharmacists’ diagnoses were evaluated by assessors and found to be accurate in over a third of cases (34%). However, assessors’ rate of disagreement with pharmacists’ diagnoses was higher. In addition, assessors found the questioning and medica history-recording practices of pharmacists to be inadequate. Pharmacists’ approaches to treatment were accurate in almost half of all cases. Over half of patients felt that their skin problem had been completely resolved through the treatment provided at the pharmacy.	Patient assessment-related education for pharmacists should be considered, with particuar focus on dermatology.

AD, atopic dermatitis; TCs, topical corticosteroids; OTC, over-the-counter; FTU, fingertip unit; TOPICOP, topical corticosteroid phobia; GP, general practitioner; CPs, compounded preparations, CAMs, complementary and alternative medicines; MUR, medicine use review.
