# Supplementary material for: Practices and Perceptions of Community Pharmacists in the Management of Atopic Dermatitis: A Systematic Review and Thematic Synthesis
Source: Healthcare (Basel). 2023 Jul 29;11(15):2159. doi: 10.3390/healthcare11152159 (PMC10418591; doi:10.3390/healthcare11152159)
Supplement: Supplementary file 1 [file healthcare-11-02159-s001.zip › Supplementary Table S1 (search strategy).pdf]

Table S1. Detailed search strategy.

Embase (1974 to 2022 December) and Medline (1946 to 2022 December) final search
1. atopic dermatitis.mp.
2. eczema.mp.
3. dermatitis.mp.
4. atopic eczema.mp.
5. corticosteroid*.mp.
6. emollient*.mp.
7. antihistamine*.mp.
8. calcineurin inhibitor*.mp.
9. 1 or 2 or 3 or 4 or 5 or 6 or 7 or 8
10. pharmacist*.mp.
11. community pharmacist*.mp.
12. pharmacy.mp.
13. 10 or 11 or 12
14. 9 and 13

Cinahl (1984 to 2022 December) final search
1. atopic dermatitis or eczema or atopic eczema or dermatitis or corticosteroid* or emollient* or antihistamine* or calcineurin inhibitor*
2. pharmacist* or community pharmacist* or pharmacy
3. S1 and S2

PsycInfo (1967 to 2022 December) final search
1. atopic dermatitis.mp. [mp=title, abstract, heading word, table of contents, key concepts, original title, tests & measures, mesh word]
2. eczema.mp. [mp=title, abstract, heading word, table of contents, key concepts, original title, tests & measures, mesh word]
3. dermatitis.mp. [mp=title, abstract, heading word, table of contents, key concepts, original title, tests & measures, mesh word]
4. atopic eczema.mp. [mp=title, abstract, heading word, table of contents, key concepts, original title, tests & measures, mesh word]
5. corticosteroid*.mp. [mp=title, abstract, heading word, table of contents, key concepts, original title, tests & measures, mesh word]
6. emollient*.mp. [mp=title, abstract, heading word, table of contents, key concepts, original title, tests & measures, mesh word]
7. antihistamine*.mp. [mp=title, abstract, heading word, table of contents, key concepts, original title, tests & measures, mesh word]
8. calcineurin inhibitor*.mp. [mp=title, abstract, heading word, table of contents, key concepts, original title, tests & measures, mesh word]
9. 1 or 2 or 3 or 4 or 5 or 6 or 7 or 8
10. pharmacist*.mp. [mp=title, abstract, heading word, table of contents, key concepts, original title, tests & measures, mesh word]
11. community pharmacist*.mp. [mp=title, abstract, heading word, table of contents, key concepts, original title, tests & measures, mesh word]
12. pharmacy.mp. [mp=title, abstract, heading word, table of contents, key concepts, original title, tests & measures, mesh word]
13. 10 or 11 or 12
14. 9 and 13

Pubmed (1972 to 2022 December) final search

Search: **("Dermatitis, Atopic"[Mesh] OR "atopic dermatitis"[tw] OR "eczema"[tw] OR "dermatitis"[tw] OR "atopic eczema"[tw] OR "corticosteroid*"[tw] OR "emollient*"[tw] OR "antihistamine*"[tw] OR "calcineurin inhibitor*" [tw]) AND ("Pharmacists"[Mesh] OR "Pharmacies"[Mesh] OR "pharmacist*"[tw] OR "community pharmacist*"[tw] OR "pharmacy"[tw])**
