# Supplementary material for: Practices and Perceptions of Community Pharmacists in the Management of Atopic Dermatitis: A Systematic Review and Thematic Synthesis
Source: Healthcare (Basel). 2023 Jul 29;11(15):2159. doi: 10.3390/healthcare11152159 (PMC10418591; doi:10.3390/healthcare11152159)
Supplement: Supplementary file 1 [file healthcare-11-02159-s001.zip › Supplementary Table S2 (data appraisal tool) (1).pdf]

**Table S2**. Quality assessment of included studies.

| Studies | Criteria from the Mixed Methods Appraisal Tool | | | | | | | | | | | | | | | | | | | | | | | | |
|---|---|---|---|---|---|---|---|---|---|---|---|---|---|---|---|---|---|---|---|---|---|---|---|---|---|
| | 1.1 | 1.2 | 1.3 | 1.4 | 1.5 | 2.1 | 2.2 | 2.3 | 2.4 | 2.5 | 3.1 | 3.2 | 3.3 | 3.4 | 3.5 | 4.1 | 4.2 | 4.3 | 4.4 | 4.5 | 5.1 | 5.2 | 5.3 | 5.4 | 5.5 |
| Abed, 2021 | | | | | | | | | | | | | | | | 0 | 0 | 0 | 0 | 1 | | | | | |
| Carr, 2009 | | | | | | | | | | | 0 | 1 | 0 | 0 | 1 | | | | | | | | | | |
| Cowdell, 2011 | 1 | 1 | 1 | 1 | 1 | | | | | | | | | | | | | | | | | | | | |
| Giua, 2021 | | | | | | | | | | | | | | | | 1 | 1 | 1 | 1 | 1 | | | | | |
| Hammarstrom, 1995 | | | | | | | | | | | 1 | 1 | 0 | 0 | 1 | | | | | | | | | | |
| Issa, 2016 | | | | | | | | | | | | | | | | 0 | 0 | 1 | 1 | 0 | | | | | |
| Jairoun, 2020 | | | | | | | | | | | | | | | | 1 | 1 | 1 | 1 | 1 | | | | | |
| Kaneko, 2014 | | | | | | | | | | | | | | | | 1 | 0 | 1 | 0 | 1 | | | | | |
| Koster, 2019 | 1 | 1 | 0 | 1 | 1 | | | | | | | | | | | | | | | | | | | | |
| Koster, 2021 | | | | | | | | | | | 1 | 1 | 1 | 0 | 1 | | | | | | | | | | |
| Lambrecht, 2019 | | | | | | | | | | | | | | | | 1 | 0 | 0 | 1 | 1 | | | | | |
| Lau, 2017 | 1 | 1 | 1 | 1 | 1 | | | | | | | | | | | 1 | 0 | 1 | 0 | 1 | 1 | 1 | 1 | 1 | 0 |
| Lindblad, 2006 | 1 | 1 | 1 | 1 | 1 | | | | | | | | | | | | | | | | | | | | |
| Manahan, 2011 | | | | | | | | | | | | | | | | 0 | 0 | 0 | 1 | 1 | | | | | |
| Oishi, 2019 | | | | | | | | | | | | | | | | 0 | 0 | 1 | 1 | 1 | | | | | |
| Raffin, 2016 | | | | | | | | | | | | | | | | 1 | 1 | 1 | 0 | 1 | | | | | |
| Salzmann, 2020 | | | | | | | | | | | | | | | | 1 | 0 | 0 | 0 | 1 | | | | | |
| Smith, 2016 | | | | | | | | | | | 0 | 0 | 1 | 0 | 1 | | | | | | | | | | |
| Teixeira, 2021 | | | | | | | | | | | | | | | | 0 | 1 | 1 | 0 | 1 | | | | | |

| | | | | | | | | | | | | | | | | | | | | | | | | | |
|---|---|---|---|---|---|---|---|---|---|---|---|---|---|---|---|---|---|---|---|---|---|---|---|---|---|
| Thandar, 2019 | | | | | | | | | | | | | | | | 1 | 0 | 1 | 0 | 1 | | | | | |
| Tucker, 2012 | | | | | | | | | | | | | | | | 1 | 0 | 1 | 0 | 1 | | | | | |
| Tucker, 2013 | | | | | | | | | | | | | | | | 1 | 0 | 1 | 0 | 1 | | | | | |
| Tucker, 2017 | 1 | 1 | 1 | 1 | 1 | | | | | | | | | | | 0 | 0 | 1 | 0 | 1 | 1 | 0 | 1 | 0 | 1 |

**Scores of studies**

| **Quantitative Studies** | **Score**$^{x}$ |
|---|---|
| Abed, 2021 | 1 |
| Carr, 2009 | 2 |
| Giua, 2021 | 5 |
| Hammarstrom, 1995 | 3 |
| Issa, 2016 | 2 |
| Jairoun, 2020 | 5 |
| Kaneko, 2014 | 3 |
| Koster, 2021 | 4 |
| Lambrecht, 2019 | 3 |
| Manahan, 2011 | 2 |
| Oishi, 2019 | 3 |
| Raffin, 2016 | 4 |
| Salzmann, 2020 | 2 |
| Smith, 2016 | 2 |
| Teixeira, 2021 | 3 |
| Thandar, 2019 | 3 |
| Tucker, 2012 | 3 |
| Tucker, 2013 | 3 |
| **Qualitative Studies** | **Score**$^{x}$ |
| Cowdell, 2019 | 5 |
| Koster, 2019 | 4 |
| Lindblad, 2006 | 5 |
| **Mixed-Methods Studies** | **Score**$^{x}$ |
| Lau, 2017 | 3 |
| Tucker, 2017 | 2 |

$^{x}$0-1= low quality; 2-3= medium quality; 4-5= high quality
