# Supplementary material for: Practices and Perceptions of Community Pharmacists in the Management of Atopic Dermatitis: A Systematic Review and Thematic Synthesis
Source: Healthcare (Basel). 2023 Jul 29;11(15):2159. doi: 10.3390/healthcare11152159 (PMC10418591; doi:10.3390/healthcare11152159)
Supplement: Supplementary file 1 [file healthcare-11-02159-s001.zip › Supplementary Table S3 (CERQual Full Evidence Profile) (1).pdf]

Supplementary Table S3. CERQual full evidence profile.

Summary of review finding	Contributing studies	Methodological limitations	Coherence	Relevance	Adequacy	CERQual assessment of confidence	Explanation of CERQual assessment
**Descriptive Themes**							
**Theme 1: Current Practice**							
**Subtheme 1: Knowledge of corticosteroids and other treatments**							
1: Pharmacists had insufficient knowledge about the potency of TCs.	[36,53,54]	*Moderate concerns*<br><br>Contributing studies show very moderate concerns due to lack of sampling strategy in two studies.	*No or very minor concerns*<br><br>There is coherence between the finding and the data in relation to low pharmacist knowledge of corticosteroid types and potency.	*Minor concerns*<br><br>Minor concerns regarding relevance. A mix of three countries only (Jordan, UAE, UK)	*Minor concerns*<br><br>Each of these studies adequately inform the review finding, all of which report on lack of knowledge of pharmacists have regarding TCs.	**High confidence**	No or very minor concerns regarding methodological limitations, coherence, and minor concerns regarding adequacy and relevance.
2: Pharmacists had lack of knowledge about the duration of TCs which should be applied.	[48,49,51,54]	*Minor concerns*<br><br>Minor concerns regarding methodological limitations due to the reliable ratings across the MMAT tool in the main studies informing this finding. Most of the studies are medium quality.	*No or very minor concerns*<br><br>The data informing this finding is coherent.	*Moderate concerns*<br><br>Studies are from across different countries and continents: Portugal, France, Belgium, Jordan, but three studies include other population, such as doctors, pharmacy technicians, etc.	*Minor concerns*<br><br>Each of these studies adequately inform the review finding, all of which report on pharmacist recommendations for the duration of TC use.	**Moderate confidence**	No or very minor concerns regarding methodological limitations and coherence, and minor concerns regarding adequacy, and moderate concerns regarding relevance)
3: Pharmacists told patients to apply	[42,48,54]	*Minor concerns*	*No or very minor concerns*	*Moderate concerns*	*Minor concerns*	**Moderate confidence**	No or very minor regarding

thinly rather than urging to use a standardized way, FTU.		Mostly medium quality reliable ratings across the MMAT tool in the studies informing this finding.	The data informing this finding is coherent, as it reports on mostly pharmacist approach applying TCs thin instead of FTU use.	Contributing studies are from three different countries (Netherlands, Portugal, Jordan) and two continents, but two studies involve different population besides pharmacists.	Each of these studies adequately inform the review finding.		methodological limitations and coherence, minor concerns regarding adequacy, moderate concerns regarding relevance.
4: Only small number of pharmacists advised the use of standardised way of topical application, FTU, during counselling.	[47,51]	*Moderate concerns*<br><br>Contributing reports had moderate concerns regarding lack of reporting clear description of target population or inclusion-exclusion criteria for participants.	*No or very minor concerns*<br><br>Minimal FTU suggestion by pharmacists well described and supported with quantitative results and the results were coherent.	*Minor concerns*<br><br>The same results from two different countries (Belgium and Japan). One study include different population besides pharmacists.	*Minor concerns*<br><br>Two studies with rich data.	**Moderate confidence**	No or very minor concerns regarding coherence, minor concerns regarding relevance and adequacy, moderate concerns regarding methodological limitations.
5: Although majority of pharmacists in studies knew FTU way, only minority of them often/always suggested patients.	[36,47]	*Moderate concerns*<br><br>Contributing reports had moderate concerns regarding lack of reporting clear description of target population or inclusion-exclusion criteria for participants.	*Minor concerns*<br><br>There is coherence between the finding and the data supporting it with regards to small number of pharmacists advising FTU use.	*Minor concerns*<br><br>Two different countries, continents and results are relevant.	*Minor concerns*<br><br>Minor concerns about adequacy (Two studies with moderately rich data overall)	**Moderate Confidence**	Minor concerns regarding coherence, relevance, adequacy, moderate concerns regarding methodological limitations.
6: Corticophobia was prevalent in pharmacists.	[36,42,43,49,51,52]	*Minor concerns*	*No or very minor concerns*	*Minor concerns*	*No or very minor concerns*	**High confidence**	No or very minor concerns regarding

		Main contributing studies show no or very minor concerns. Just one study was assessed as having moderate concerns regarding methodological limitations.	It was obvious the pharmacist corticophobia in all studies	Minor concerns regarding relevance. Studies from five different countries (United Kingdom, France, Belgium, Italy, Netherlands) contributed to this finding, and the finding is relevant to research question and aim, but the participants of some studies were not only pharmacists.	Six studies with rich data contributed to the finding.		coherence and adequacy, minor concerns regarding relevance and methodological limitations.
7: Corticophobia was higher in pharmacists than other healthcare professionals.	[51]	*Moderate concerns*<br><br>Only one contributing study had moderate concerns in relation to representation of target population.	*No or very minor concerns*<br><br>No or very minor concerns are raised	*Serious concerns*<br><br>Only one study from Belgium contributed to the finding.	*Serious concerns*<br><br>Only one study represents the finding.	**Low confidence**	No or very minor concerns regarding coherence, minor concerns regarding methodological limitations, serious concerns regarding relevance and adequacy.
8: The confidence of pharmacists about the use of TCs was also medium.	[49]	*Minor concerns*<br><br>Minor concerns because of low response rate to contributing study.	*No or very minor concerns*<br><br>No or very minor concerns about coherence	*Serious concerns*<br><br>Only one study from France contributed to the finding.	*Serious concerns*<br><br>Only one study from France offering data	**Low confidence**	No or very minor concerns regarding coherence, minor concerns regarding methodological limitations, serious concerns regarding relevance and adequacy

9: The differentiate between side effects of oral and TCs was mingled by pharmacists.	[49,51]	*Moderate concerns*<br><br>Moderate concerns regarding methodological limitations were identified because of low response rate in one study and low representativeness in another study.	*Moderate concerns*<br><br>Data varied and the finding was measured as secondary outcome.	*Moderate concerns*<br><br>Partial relevance because the data was only from two countries and presented as a secondary outcome.	*Minor concerns*	**Moderate confidence**	Minor concerns regarding adequacy, moderate concerns regarding coherence, relevance, methodological limitations.
10: Regular use of emollient was recommended as a first-line treatment by pharmacists.	[36,54]	*Moderate concerns*<br><br>Moderate concerns regarding methodological limitations were determined due to undescribed inclusion and exclusion criteria for recruitment in all studies, and inadequate sampling strategy in one study.	*No or very minor concerns*<br><br>There is coherence between the finding and the data in relation to emollient recommendation by pharmacists.	*Moderate concerns*<br><br>Data more varied and this finding is just one of the findings among many others in the studies.	*Minor concerns*<br><br>Minor concerns about adequacy (Two studies with moderately rich data overall)	**Moderate confidence**	No or very minor concerns regarding coherence, minor concerns regarding adequacy, moderate concerns regarding methodological limitations and relevance.
11: Even if patients were not prescribed an emollient by doctor, it was recommended by pharmacists.	[42]	*No or very minor concerns*<br><br>No or very minor concerns regarding methodological limitations.	*No or very minor concerns*<br><br>No or very minor concerns about coherence	*Serious concerns*<br><br>Only one study from Netherlands contributing to the finding.	*Serious concerns*<br><br>Only one study offering data.	**Low confidence**	No or very minor concerns regarding coherence and methodological limitations, serious concerns regarding relevance and adequacy.
12: Emollients were advised to be used regularly and for long-time period.	[36]	*Minor concerns*<br><br>Minor concerns regarding	*No or very minor concerns*	*Serious concerns*<br><br>Only one study from United Kingdom	*Serious concerns*<br><br>Only one study offering data.	**Low confidence**	No or very minor concerns regarding coherence, minor

		methodological limitations were determined.	No or very minor concerns about coherence	contributing to the finding.			concerns regarding methodological limitations, serious concerns regarding relevance and adequacy.
13: CAMs use was perceived more useful than conventional therapy for AD by pharmacists.	[55]	*Moderate concerns*<br><br>Moderate concerns regarding data sampling and high non-response rate.	*No or very minor concerns*<br><br>No or very minor concerns about coherence	*Serious concerns*<br><br>Only one study from South Africa contributing to the finding.	*Serious concerns*<br><br>Only one study offering data.	**Low confidence**	No or very minor concerns regarding coherence, moderate concerns regarding methodological limitations, serious concerns regarding relevance and adequacy.
14: Tingling was given as a common side effect of TCIs by pharmacists while counselling patients, but it was nothing to concern much.	[46]	*Moderate concerns*<br><br>Moderate concerns regarding data sampling and high non-response rate.	*Minor concerns*<br><br>Varied primary data presented.	*Serious concerns*<br><br>Only one study from Japan contributing to the finding.	*Serious concerns*<br><br>Only one study offering data.	**Low confidence**	Minor concerns regarding coherence, moderate concerns regarding methodological limitations, serious concerns regarding relevance and adequacy.
15: There was lack of recommendation about lifestyle among pharmacists.	[42]	*No or very minor concerns*<br><br>No or very minor concerns regarding	*No or very minor concerns*	*Serious concerns*<br><br>Only one study from Netherlands	*Serious concerns*<br><br>Only one study offering data.	**Low confidence**	No or very minor concerns regarding coherence and methodological

		methodological limitations.	No or very minor concerns about coherence	contributing to the finding.			limitations, serious concerns regarding relevance and adequacy.
**Subtheme 2: The frequency and diagnosis of Atopic Dermatitis in pharmacies**							
16: AD was seen one of the most common skin conditions in pharmacies.	[37,39,40,48]	*Serious concerns*<br><br>Serious concerns with regards to sampling strategy, representativeness of sample, inappropriate measurement in one study, and non-response risk in three studies.	*Minor concerns*<br><br>Varied primary data presented and the finding is relevant to primary data.	*Minor concerns*<br><br>Minor concerns regarding relevance. Studies from three different countries and continents (United Kingdom, Australia, Portugal) contributed to this finding, and the finding is relevant to research question and aim, but the participants of one study were not only pharmacists.	*Minor concerns*<br><br>Four studies with enough data contributed to the finding.	**Moderate confidence**	Minor concerns regarding relevance, coherence, and adequacy, serious concerns regarding methodological limitations.
17: Pharmacists were doing more medicine use review for AD than other skin conditions, such as acne, psoriasis.	[39]	*Moderate concerns*<br><br>Moderate concerns regarding methodology limitations with lack of explanation how sample group was determined and lack of response rate.	*No or very minor concerns*<br><br>No or very minor concerns about coherence	*Serious concerns*<br><br>Only one study from United Kingdom contributing to the finding.	*Serious concerns*<br><br>Only one study offering data.	**Low confidence**	No or very minor concerns regarding coherence, moderate concerns regarding methodological limitations, serious concerns regarding relevance and adequacy.
18: Dermatologists were on agreement with some diagnosis	[38]	*Moderate concerns*	*Minor concerns*	*Serious concerns*	*Serious concerns*	**Low confidence**	Minor concerns regarding coherence,

by pharmacists, but the history taking was low.		The inclusion and exclusion of sample group was not detailed, and non-response bias should be taken into consideration.	Varied primary data presented.	Only one study from United Kingdom contributing to the finding and data more varied between AD an acne.	Only one study offering data.		moderate concerns regarding methodological limitations, serious concerns regarding relevance and adequacy.
19: Dermatologist only agreed on small number of AD cases where treatment options were provided by pharmacists.	[40]	*Moderate concerns*<br><br>It was not clear if sampling strategy was relevant to address the research question, the inclusion and exclusion of sample group was not detailed.	*No or very minor concerns*<br><br>No or very minor concerns about coherence	*Serious concerns*<br><br>Only one study from Australia contributing to the finding	*Serious concerns*<br><br>Only one study offering data.	**Low confidence**	No or very minor concerns regarding coherence, moderate concerns regarding methodological limitations, serious concerns regarding relevance and adequacy.
**Subtheme 3: Continuing training for pharmacists**							
20: It was shown that many pharmacists continued to get postgraduate dermatology education.	[36,37,39,40,49,52,53,55]	*Moderate concerns*<br><br>*Although two studies were with no concerns,* the inclusion and exclusion of sample group was not detailed, and non-response bias should be taken into consideration in four studies.	*Minor concerns*<br><br>Varied primary data presented and the finding is relevant to primary data.	*Minor concerns*<br><br>Minor concerns regarding relevance. Studies from six different countries and continents (United Kingdom, Australia, South Africa, UAE, Italy, France) contributed to this finding, and the finding is relevant to research question and aim, but the participants of	*No or very minor concerns*<br><br>Eight studies with enough data contributed to the finding.	**Moderate confidence**	No or very concerns regarding adequacy, minor concerns regarding coherence and relevance, moderate concerns regarding methodological limitations.

				three studies were not only pharmacists.			
21: Pharmacists were interested in extending their dermatological knowledge by attending different programs.	[35,36,37,49]	*Minor concerns*<br><br>Low response rate in three studies, but qualitative and mixed method studies had high quality regarding methodology.	*No or very minor concerns*<br><br>No or very minor concerns about coherence	*Moderate concerns*<br><br>Data more varied and this finding is just one of the findings among many others in the studies. Data from two different countries contributed to the finding.	*Minor concerns*<br><br>Four studies with enough data contributed to the finding.	**Moderate confidence**	No or very minor concerns regarding coherence, minor concerns regarding methodological limitations and adequacy, moderate concerns regarding relevance.
22: Pharmacists continuing training in dermatology were more confident and these training helped them a lot.	[39]	*Moderate concerns*<br><br>Moderate concerns regarding methodology limitations with lack of explanation how sample group was determined and lack of response rate.	*No or very minor concerns*<br><br>No or very minor concerns about coherence	*Serious concerns*<br><br>Only one study from United Kingdom contributing to the finding.	*Serious concerns*<br><br>Only one study offering data.	**Low confidence**	No or very minor concerns regarding coherence, moderate concerns regarding methodological limitations, serious concerns regarding relevance and adequacy.
23: Even pharmacists who got continuing training in dermatology displayed better practice.	[53]	*No or very minor concerns*<br><br>No or very minor concerns regarding methodological limitations.	*No or very minor concerns*<br><br>No or very minor concerns about coherence	*Serious concerns*<br><br>Only one study from UAE contributing to the finding.	*Moderate concerns*<br><br>Only one study offering data, but it had enough data.	**Moderate confidence**	No or very minor concerns regarding coherence and methodological limitations, serious concerns regarding relevance and adequacy.

**Theme 2: Impact of pharmacists**							
**Subtheme 1: Outcomes between pharmacists and patients**							
24: Pharmacies were accepted as first point to come with a skin problem.	[37,45,52,54]	*Moderate concerns*<br><br>It was not clear if sampling strategy was relevant to address the research question in a study, and the inclusion and exclusion of sample group was not detailed in two studies.	*No or very minor concerns*<br><br>No or very minor concerns about coherence	*Minor concerns*<br><br>Data more varied and this finding is just one of the findings among many others in the studies. Data from four different countries contributed to the finding.	*Minor concerns*<br><br>Four studies with enough data contributed to the finding.	**High confidence**	No or very minor concerns regarding coherence, minor concerns regarding relevance and adequacy, moderate concerns regarding methodological limitations.
25: Patients got referred to doctors when the condition got worse.	[45,54]	*Moderate concerns*<br><br>It was not clear if sampling strategy was relevant to address the research question, the inclusion and exclusion of sample group was not detailed.	*No or very minor concerns*<br><br>No or very minor concerns about coherence	*Minor concerns*<br><br>Varied primary data presented and the finding is relevant to primary data.	*Moderate concerns*<br><br>One study with enough data.	**Moderate confidence**	Minor concerns regarding relevance, moderate concerns regarding methodological limitations, coherence and adequacy.
26: Patients mostly did not get any recommendation on the use of TCs by pharmacists in Iraq.	[56]	*Serious concerns*<br><br>The contributing study was rated low regarding methodological quality.	*Minor concerns*<br><br>The finding was coherent with data.	*Serious concerns*<br><br>Only one study from Iraq contributing to the finding.	*Serious concerns*<br><br>Only one study offering data.	**Very low confidence**	Minor concerns regarding coherence, serious concerns regarding methodological limitations, relevance, and adequacy.
27. Some patients were uncomfortable when the counselling was	[45]	*No or very minor concerns*	*No or very minor concerns*	*Serious concerns*	*Serious concerns*<br><br>Only one study offering data.	**Low confidence**	No or very minor concerns regarding coherence and

happening in front of other patients, and they claimed pharmacists are not understanding patient circumstance.		No or very minor concerns regarding methodological limitations.	No or very minor concerns about coherence	Only one study from Sweden contributing to the finding.			methodological limitations, serious concerns regarding relevance and adequacy.
**Subtheme 2: Inter-professional communication**							
28: There was weak communication and cooperation between pharmacists and healthcare professionals	[35,45,49,50]	*Minor concerns*<br><br>Two qualitative studies had no concerns, but other two had some concerns with moderate non-response rate.	*Minor concerns*<br><br>The finding was coherent with data.	*Moderate concerns*<br><br>Although data from four countries (United Kingdom, Sweden, Germany, France) contributed, but some data covered many skin conditions, not only AD.	*Minor concerns*<br><br>Four studies with enough data contributed to the finding.	**Moderate confidence**	Minor concerns regarding methodological limitations, coherence and adequacy, moderate concerns regarding relevance.
29: Different guidelines may be followed by healthcare professionals, so this may make patients confused when they get counselled by pharmacists and other healthcare workers.	[35]	*No or very minor concerns*<br><br>No or very minor concerns regarding methodological limitations.	*No or very minor concerns*<br><br>No or very minor concerns about coherence	*Serious concerns*<br><br>Only one study from United Kingdom contributing to the finding.	*Serious concerns*<br><br>Only one study offering data.	**Low confidence**	No or very minor concerns regarding coherence and methodological limitations, serious concerns regarding relevance and adequacy.
30: The length of time advised by doctor, at which TCs should be applied by patients, changed by pharmacists even if it was not often seen.	[49,50]	*Moderate concerns*<br><br>Moderate concerns regarding methodological limitations were identified because of low response rate in	*Minor concerns*<br><br>The finding was coherent with data.	*Moderate concerns*<br><br>Although data from two countries (Germany, France) contributed, but one study covered other	*Minor concerns*<br><br>Two studies with enough data contributed to the finding.	**Moderate confidence**	Minor concerns regarding coherence and adequacy, moderate concerns regarding methodological

		two studies and low representativeness in another study.		skin conditions, not only AD.			limitations and relevance.
